# Primary hepatic neuroendocrine carcinoma: report of two cases and literature review

**DOI:** 10.1186/s12907-018-0070-7

**Published:** 2018-03-01

**Authors:** Zi-Ming Zhao, Jin Wang, Ugochukwu C. Ugwuowo, Liming Wang, Jeffrey P. Townsend

**Affiliations:** 1 The Jackson Laboratory for Genomic Medicine, 10 Discovery Drive, Farmington, CT 06032 USA; 2 Department of Biostatistics, Yale University, New Haven, CT 06511 USA; 3 Division of Vascular Surgery, The Second Affiliated Hospital of Dalian Medical University, Dalian, Liaoning Province 116000 China; 4 Division of Hepatobiliary and Pancreatic Surgery, The Second Affiliated Hospital of Dalian Medical University, 467 Zhongshan Road, Dalian, Liaoning Province 116000 China; 5 Dalian Medical University, Dalian, Liaoning Province 116000 China; 6 Department of Epidemiology of Microbial Diseases, Yale School of Public Health, New Haven, CT 06520 USA; 7 Suite 200, 135 College St, New Haven, CT 06510 USA; 8 Division of Hepatobiliary and Pancreatic Surgery, Department of General Surgery, The Second Affiliated Hospital of Dalian Medical University, 467 Zhongshan Road, Dalian, Liaoning 116027 China

**Keywords:** Primary hepatic neuroendocrine carcinoma, Immunohistochemical staining, Liver biopsy, Transcatheter arterial chemoembolization, Somatostatin analogue

## Abstract

**Background:**

Primary hepatic neuroendocrine carcinoma (PHNEC) is extremely rare. The diagnosis of PHNEC remains challenging—partly due to its rarity, and partly due to its lack of unique clinical features. Available treatment options for PHNEC include surgical resection of the liver tumor(s), radiotherapy, liver transplant, transcatheter arterial chemoembolization (TACE), and administration of somatostatin analogues.

**Case presentation:**

We report two male PHNEC cases and discuss the diagnosis and treatment options. Both cases presented with abdominal pain; case two also presented with symptoms of jaundice. The initial diagnosis for both cases was poorly differentiated grade 3 small-cell neuroendocrine carcinoma, based on imaging characteristics and the pathology of liver biopsies. Final diagnoses of PHNEC were arrived at by ruling out non-hepatic origins. Case one presented with a large tumor in the right liver lobe, and the patient was treated with TACE. Case two presented with tumors in both liver lobes, invasions into the left branch of hepatic portal vein, and metastasis in the hepatic hilar lymph node. This patient was ineligible for TACE and was allergic to the somatostatin analogue octreotide. This limited treatment options to supportive therapies such as albumin supplementation for liver protection. Patient one and two died at 61 and 109 days, respectively, following initial hospital admission.

**Conclusions:**

We diagnosed both cases with poorly differentiated grade 3 small-cell PHNEC through imaging characteristics, immunohistochemical staining of liver biopsies, and examinations to eliminate non-hepatic origins. Neither TACE nor liver protection appeared to significantly extend survival time of the two patients, suggesting these treatments may be inadequate to improve survival of patients with poorly differentiated grade 3 small-cell PHNEC. The prognosis of poorly differentiated grade 3 small-cell PHNEC is poor due to limited and ineffective treatment options.

**Electronic supplementary material:**

The online version of this article (10.1186/s12907-018-0070-7) contains supplementary material, which is available to authorized users.

## Background

Primary hepatic neuroendocrine carcinoma (PHNEC) is a rare neuroendocrine carcinoma (NEC) that originates from the liver, whereas the vast majority NECs in the liver are the result of metastases that arise from the gastrointestinal tract and lungs [1–6]. Symptoms of PHNEC are not specific, and patients normally present with abdominal pain [7]. Thus, diagnosis of PHNEC is very challenging. Liver cancer is commonly diagnosed initially, typically relying on imaging methods such as ultrasound, enhanced computer tomography (CT), and dynamic contrast-enhanced magnetic resonance imaging (DCE-MRI). A diagnosis of NEC then results from pathological examination of pre-operative liver tumor biopsy or the surgically resected tumor, and a final diagnosis of PHNEC largely depends on ruling out non-hepatic origins [7–9]. Treatment of PHNEC includes surgical resection of liver (partial hepatectomy), transcatheter arterial chemoembolization (TACE) therapy, liver transplantation, chemotherapy, radiotherapy or radiofrequency ablation, and administration of somatostatin (i.e., growth hormone-inhibiting hormone) analogues, with the choice of treatment depending on tumor stage, location of the tumor in the liver, and whether the carcinoma secretes hormones [7]. Partial hepatectomy is the most common used treatment, particularly for localized PHNECs [8, 9], while TACE is normally performed for advanced PHNEC cases that are poor candidates for resection [7, 10]. By interrupting the blood supply to the tumor while directly delivering chemotherapeutics, TACE inhibits tumor growth and extends survival. Somatostatin analogues, such as octreotide, can inhibit secretion of growth hormone by NECs and prevent tumor proliferation [11].

Here, we report two PHNEC cases that were diagnosed and treated at our hospital. Since PHNEC is extremely rare and lacks specific diagnostic symptoms, diagnosis of each case proceeded through several important stages of examination. Imaging methods (DCE-MRI and enhanced CT) revealed a mass in the liver, supporting an initial diagnosis of liver cancer. Subsequent immunohistochemical (IHC) examination of liver biopsies confirmed the diagnosis of poorly differentiated NEC. Finally, the primary hepatic origin of the detected NEC in each case was arrived at when gastroscopy, colonoscopy, and positron emission tomography-computer tomography (PET-CT) examinations eliminated the possibility of the NEC having metastasized to the liver from elsewhere in the body. We present discussion of the diagnosis, pathology, treatment, and prognosis of PHNEC.

## Case presentation

### Case one

**Fig. 1:**
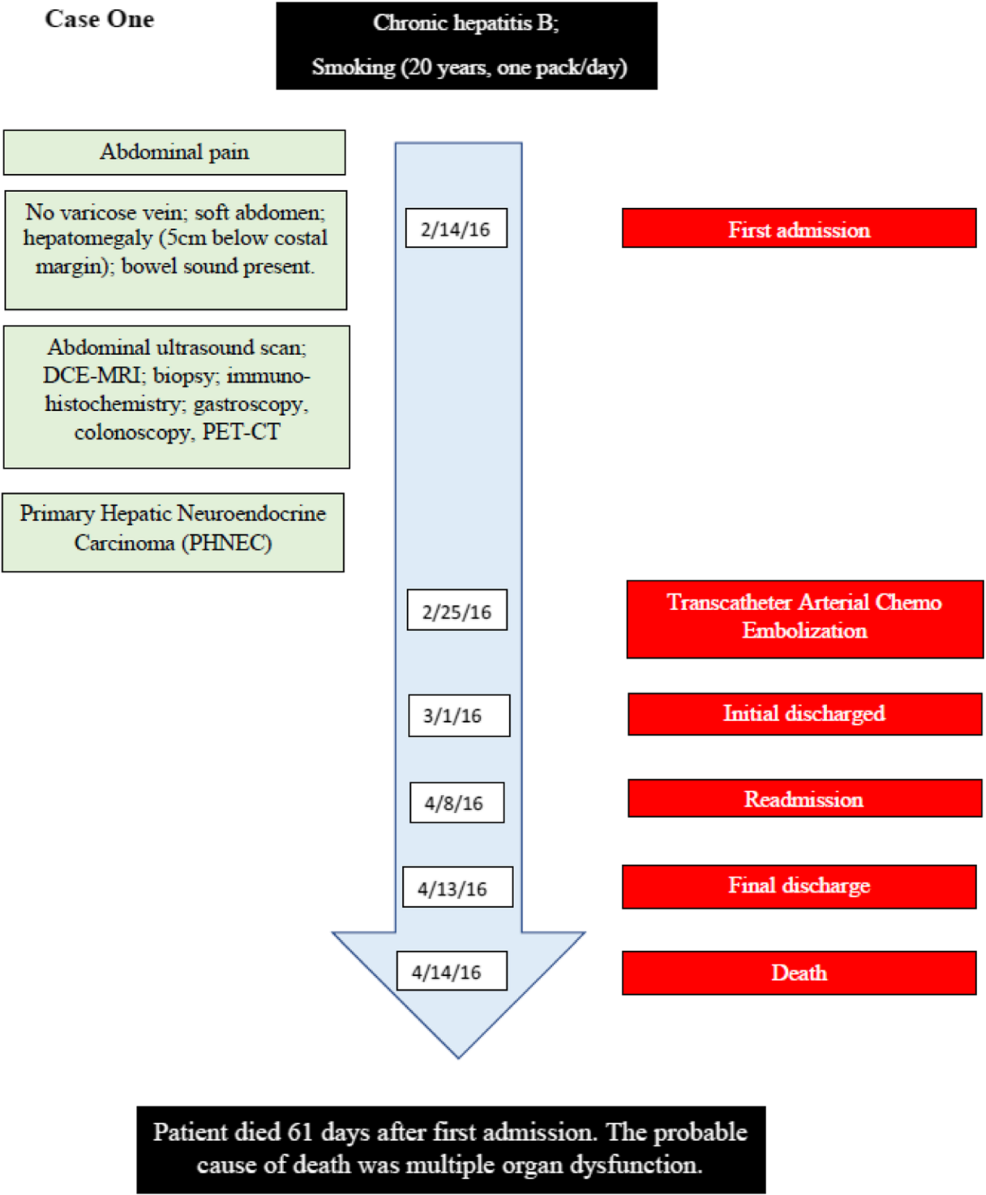
Case one timeline

A 61-year-old retired Chinese male was admitted into our hospital in February 2016, presenting with upper abdominal pain for over 1 month (Fig. 1). Liver lesions were found via ultrasound in another hospital 2 weeks after symptoms appeared. The patient had a history of chronic hepatitis B and 20 years of smoking one pack per day. On inspection, there were no varicose veins; on palpation, the abdomen was soft, and non-tender; on percussion, hepatomegaly was detected 5 cm below the costal margin, and there was no shifting dullness that would suggest ascites; and on auscultation, bowel sounds were present (4–5/min). Serum studies indicated high alpha-fetoprotein (AFP, 878.60 IU/ml, normal range 0.00–6.70), neuron-specific enolase (NSE, 25.51 μg/L, normal range 0.00–13.00), cancer antigen 125 (CA–125, 107.83 U/mL, normal range 0.00–35.00), and serum ferritin (SF, 519.55 μg/L, normal range 0.00–322.00).

**Fig. 2:**
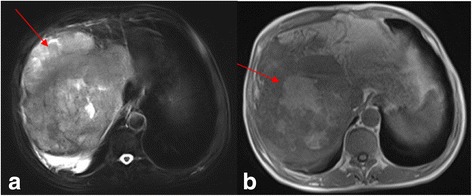
Transverse DCE-MRI of the abdomen shows lesions (arrows) in the right lobe of liver. A large mass in the right hepatic lobe is indicated by heterogeneous areas of high signal intensity (arrow) in (**a**) the T2-weighted image, and heterogeneous areas of low intensity signal (arrow) in (**b**) the T1-weighted image

**Fig. 3:**
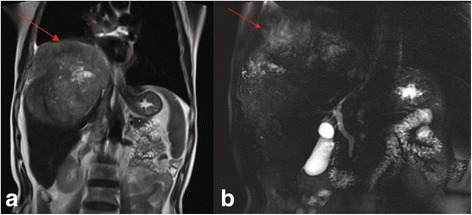
Coronal DCE-MRI of the abdomen shows liver lesions alongside normal bile ducts. **a** Localized lesions were prominent in the liver capsule, and the right diaphragmatic muscle was raised (arrow). **b** No dilation of the intrahepatic or extrahepatic bile ducts was observed (arrow)

**Fig. 4:**
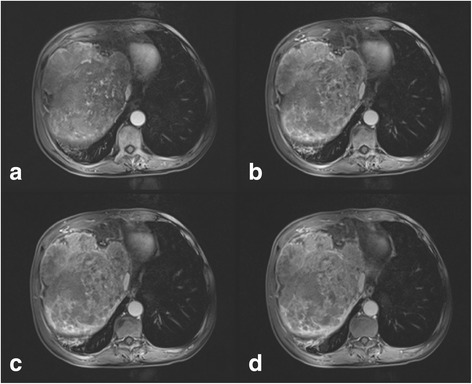
Transverse DCE-MRI of the abdomen shows a large lesion in the right lobe of the liver in four different phases. DCE-MRI showed heterogeneity of signal enhancement in the arterial phase (**a**), a more obvious heterogeneous signal enhancement in the venous phase (**b**), slightly weakened heterogeneous enhancement in the equilibrium phase (**c**), and a significantly weakened heterogeneous enhancement in the delayed phase (**d**)

**Fig. 5:**
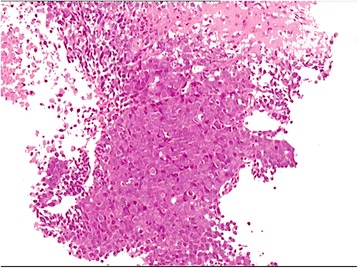
Microscopic finding of a carcinoma of neuroendocrine cells in the liver biopsy from case one. Tumor cells exhibit invasive growth with non-distinct cell borders, light-staining or basophilic cytoplasm, large and dark nuclei with an irregular shape, visible pathologic mitosis, and coagulative necrosis. The biopsy section was stained with hematoxylin-eosin and imaged at 400×. The image was adjusted in Photoshop to remove the pink background and increase the contrast, and the original image before adjustment is provided in Additional file 1: Figure S1

Abdominal ultrasound revealed a 12 × 11 cm hyperechoic mass in the right lobe of the liver. DCE-MRI of the upper abdomen revealed a large mass (17 × 14 × 14 cm) in the right lobe of the liver, as indicated by a region of mixed signal intensity (Figs. 2, 3, 4). DCE-MRI showed an enhanced intensity in the arterial phase, and a rapid decrease in the intensity of enhancement in the hepatic parenchymal phase, which supported a diagnosis of liver cancer (Fig. 4). To confirm the initial diagnosis of liver cancer, we performed an ultrasound-guided liver biopsy 1 week after admission. Pathological examination of the tissue confirmed a carcinoma of neuroendocrine cells based on IHC staining (Fig. 5 and Additional file 1: Figure S1). Specifically, the tumor was positive for: cytokeratin AE1/AE3; the neuroendocrine markers CD56 (also known as neural cell adhesion molecule) and synaptophysin (Syn); and weakly positive for the gastrointestinal adenocarcinoma marker villin and the hepatocellular carcinoma marker HepPar-1. Furthermore, 70% of the tumor cells were positive for the proliferative marker Ki-67 (i.e., IHC measured a Ki-67 index of 70%), indicating cells are actively dividing. Thus, IHC examination of the liver biopsy confirmed the diagnosis of poorly differentiated NEC (grade 3), a type of small-cell carcinoma, of uncertain origin. We further investigated the primary origin of the NEC by fully examining the patient using gastroscopy, colonoscopy and PET-CT, which failed to find NEC in any other organ. Therefore, we diagnosed the patient with PHNEC.

For treatment, 11 days after presentation, we performed TACE and delivered chemotherapuetics (total dose oxaliplatin 100 mg, camptothecin 10 mg, and pirarubicin 20 mg) and side effect-mitigating agents (total dose tropisetron 5 mg and dexamethasone 10 mg) along with the procedure. One day before TACE, a six-day course of supplementary intravenous human albumin 20% (20 g per l00 ml, 100 ml administered per day) was initiated as supportive therapy to protect the liver. We chose not to perform surgical resection due to the large size of the tumor and the overall poor physical condition of this patient. After 1 month of TACE, DCE-MRI showed partial tumor necrosis, no increase in the tumor size, and no new lesions. Two weeks later, the patient was re-admitted into our hospital and the diagnosis of decompensated cirrhosis and hepatic encephalopathy were made. The patient died six days after re-admission (61 days after first admission; Fig. 1). The probable cause of death was multiple organ dysfunction syndrome.

### Case two

**Fig. 6:**
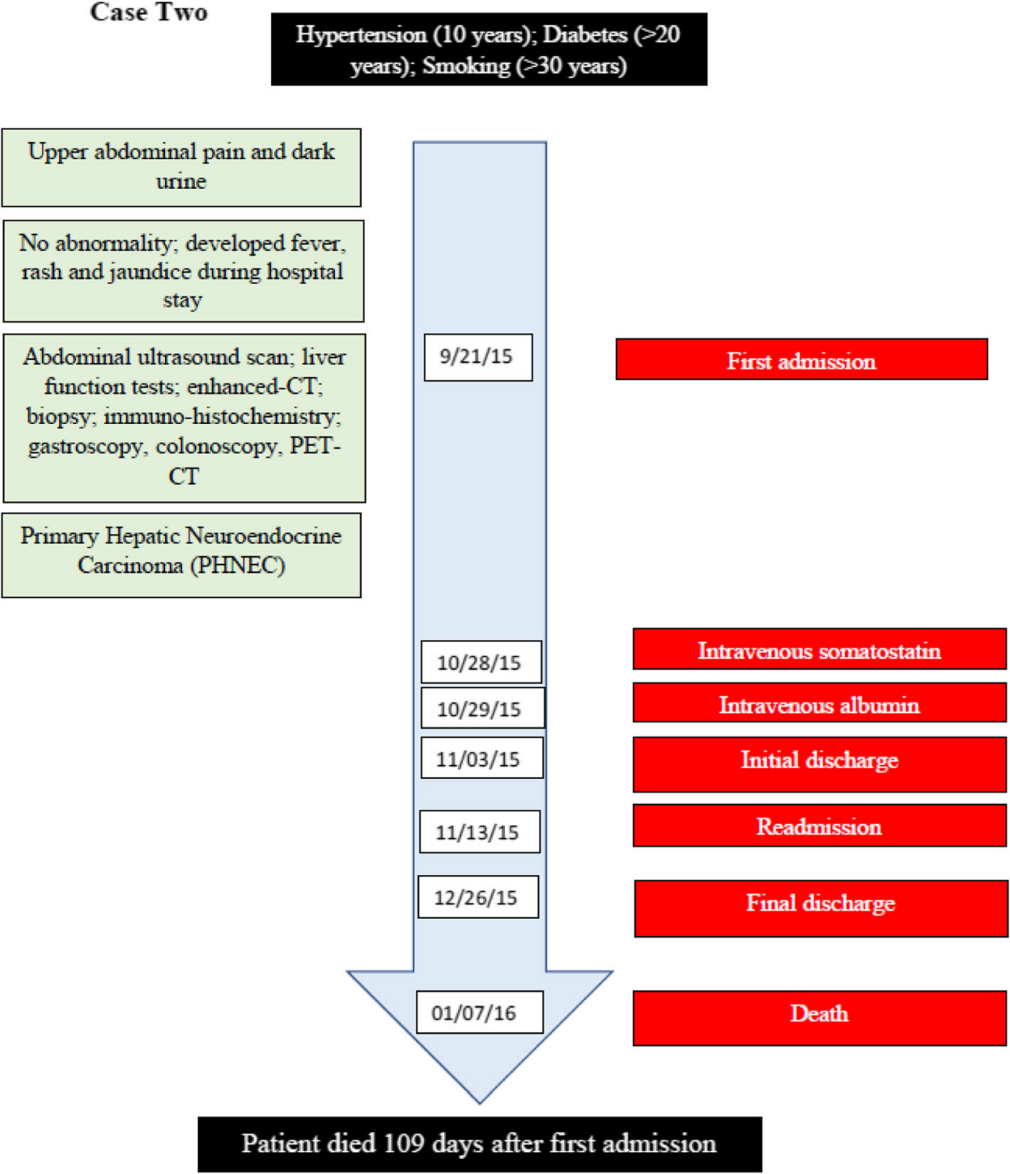
Case two timeline

A 69-year-old retired Chinese male was admitted into our hospital in September 2015, presenting with upper abdominal pain for the previous month and dark urine for the previous 2 days (Fig. 6). The patient had hypertension for more than 10 years and diabetes for more than 20 years, and he had smoked for more than 30 years. Physical examination of the abdomen showed no abnormalities. Serum studies indicated high alanine aminotransferase (ALT, 151 U/L, normal range 9–50), serum aspartate aminotransferase (AST, 68 U/L, normal range 15–40), total bilirubin (TB, 36.57 μmol/L, normal range 2.00–20.00), cancer antigen 19–9 (CA19–9, 160.46 U/ml, normally range 0.00–37.00).

Abdominal ultrasound revealed a solid liver mass, hypoechoic areas in the left branch of hepatic portal vein suggesting portal vein invasion by the liver mass, and dilation of the intrahepatic bile duct in the lateral segment of the left hepatic lobe suggesting obstruction of the bile duct. These findings suggested a diagnosis of liver cancer with portal vein invasion and obstruction of the bile duct.

**Fig. 7:**
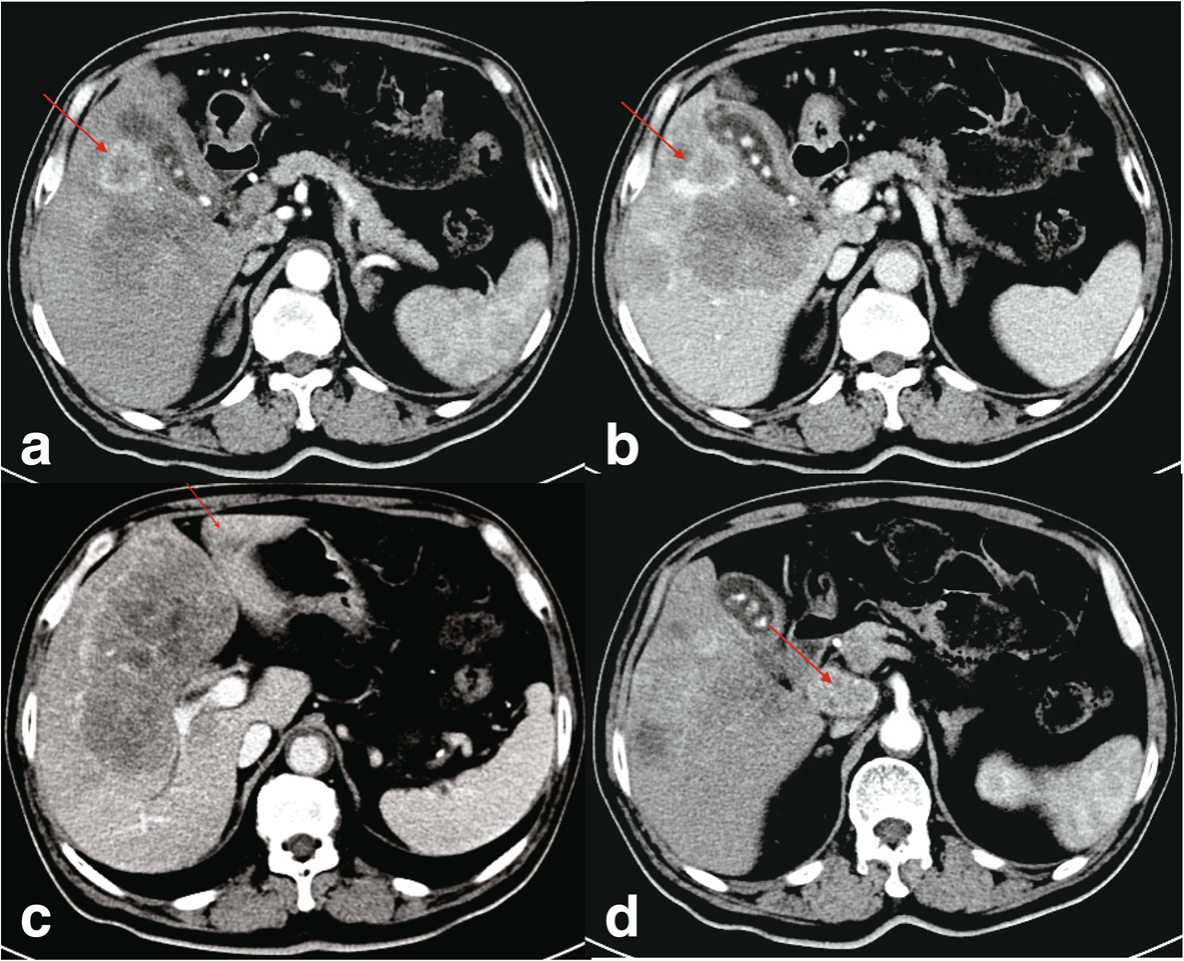
Enhanced-CT shows multiple lesions in the right and left hepatic lobes in different phases. The right hepatic lobe showed multiple patchy shadows (the largest 94 × 151 mm) and a circular enhanced lesion (arrow) in the (**a**) arterial and (**b**) venous phases. The left hepatic lobe showed a patchy shadow within the left lobe of liver (arrow) in (**c**) the venous phase. The portal triad showed an oval shadow (22 × 38 mm) with soft tissue density (arrow) in (**d**) the arterial phase

Enhanced-CT revealed multiple lesions in the right and left hepatic lobes, and invasion of the lymph node in the portal triad (Fig. 7). Multiple masses were evident in the right hepatic lobe (Fig. 7a, b), a small and patchy shadow was evident in the left hepatic lobe (Fig. 7c), and an oval shadow (22 × 38 mm) was evident in the portal triad, indicating abnormal soft tissue density (Fig. 7d). Enhanced-CT also suggested an invasion of the left portal vein and mild dilation of the bile duct in the right hepatic lobe. In summary, enhanced-CT suggested the diagnosis of intrahepatic malignant tumors, lymph node metastasis in the portal triad, and invasion of the left portal vein.

**Fig. 8:**
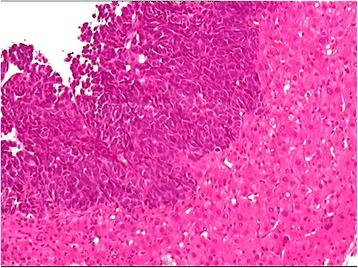
Microscopic finding of a carcinoma of neuroendocrine cells in the liver biopsy from case two. Tumor cells are clustered and composed of small cells with little cytoplasm, a high nucleo-cytoplasmic ratio, and dark nuclei with an irregular shape. The biopsy section was stained with hematoxylin-eosin and imaged at 400×. The image was adjusted in Photoshop to remove the pink background and increase the contrast, and the original image before adjustment is provided in Additional file 2: Figure S2

During the hospital stay, the patient presented with yellow skin and sclera, as well as fever and rash, suggesting the possibility of autoimmune liver disease. However, pathological examination confirmed a diagnosis of NEC (Fig. 8 and Additional file 2: Figure S2). IHC staining indicated that the cells were positive for AE1/AE3, Syn, chromogranin A (CgA) and CD56; and the IHC measured a Ki-67 index of 95%. Thus, IHC examination of the liver biopsy excluded the possibility of autoimmune liver disease and confirmed the diagnosis of poorly differentiated NEC (grade 3), a type of small-cell carcinoma, of uncertain origin. To confirm the hepatic primary origin of NEC as opposed to hepatic metastases, we performed gastroscopy, colonoscopy, and PET-CT examinations, and failed to find NEC in other organs. Therefore, we diagnosed the patient with PHNEC.

To investigate the causes for the yellow hue of the skin and sclera, we examined liver biochemical factors, measurement of which confirmed obstructive jaundice, indicated by an elevated total bilirubin level (336.97 μmol/L, normal range 2.00–20.00) with an increased level of direct bilirubin (279.76 μmol/L, normal range 0.00–6.00) and a normal level of indirect bilirubin. Obstructive jaundice was also consistent with the dilation of the bile duct that had been revealed by enhanced-CT.

For treatment, we chose not to perform surgical resection, considering the poor physical condition of the patient and the extensive spread of the tumor. We further excluded TACE treatment, because of the extent of the tumor lesions in both the right and left hepatic lobes, as well as the left portal vein. While TACE stops tumor growth by interrupting the blood supply to the tumor, normal liver cells can continue to survive relying on the portal vein. Thus, portal vein invasion contraindicated use of TACE, which would have a high risk of inducing liver failure. We treated the patient with the somatostatin analogue octreotide intravenously for 24 h at the dose of 125 μg/h, with the goal of inhibiting the growth of the neuroendocrine tumor and extending the patient’s survival. However, the patient developed itchy red rashes, which are characteristic of an allergic reaction to octreotide. Therefore, we discontinued this treatment immediately. At this point, treatment was limited to protecting the liver by a four-day course of supplementary intravenous human albumin 20% (20 g per l00 ml, 50 ml administered per day). The patient died 109 days after first admission (Fig. 6).

## Discussion

Primary liver cancers are usually hepatocellular carcinoma (HCC) and rarely neuroendocrine carcinoma, as NEC normally metastasizes to the liver rather than originating from it [12–15]. Differential diagnosis of PHNEC versus metastatic hepatic NEC is important to guide treatment options and predict outcomes [16–19]. The two cases reported here were both diagnosed as grade 3 poorly differentiated PHNEC.

PHNEC shows no gender predominance [6, 20] and no risk factors have been reported. A review of PHNEC cases estimated a mean age of 49.8 (standard deviation: ±16.0) at the time of diagnosis and that 58.5% (55/94; confidence interval, CI, 47.9%–68.6%) of patients were female [7]. In contrast, another literature review estimated a median age of 66.5 (range: 37–80) and 58.3% (7/12; CI 27%–84%) male patients [1]. Both cases we presented here were male with a mean age of 65 (61 and 69) and had been smokers for at least 20 years; case one had chronic hepatitis B, and case two had a long history of hypertension and diabetes.

PHNEC is normally detected at a late stage when the tumor is large, because patients normally present non-specific clinical symptoms. A review showed that 44% (37/84; CI 33.2%–55.3%) of patients presented abdominal discomfort, 13.1% (11/84; CI 6.8%–22.2%) of patients exhibited no symptoms, and 4.8% (4/84; CI 1.3%–11.8%) had jaundice [7]. No standard markers have been reported to be unique for PHNEC [14]. Both cases we reported here presented with abdominal pain, and serum studies showed abnormal tumor markers of AFP, NSE, CA–125, and SF for case one and CA19–9 for case two.

We diagnosed both patients as having hepatic NEC using imaging and pathological examination of biopsies. Based on imaging alone, it is challenging to distinguish hepatic NEC from other hepatic carcinomas such as HCC or distinguish primary hepatic from metastatic hepatic NEC [21]. However, some hepatic NECs exhibit distinctive cyst-like changes in the liver on MRI and CT images that can differentiate hepatic NEC from HCC [1, 2, 15, 22–25]. Retrospective investigation showed that both of our cases presented cyst-like changes in the liver that are similar to those described in the literature, which suggested a diagnosis of neuroendocrine carcinoma in the liver. With respect to pathological examination of the biopsies, IHC markers are effective for identification of primary hepatic neuroendocrine tumors, and positive staining for Syn and CgA has been used to support diagnosis of PHNEC [15, 25–27]. A review reported that 84% (26 out of 31) of PHNEC cases showed positive staining for CgA [13]. Another review reported that 95% (90 out of 95) of PHNEC cases showed positive staining for CgA [6]. For the two cases we reported here, case one exhibited positive staining for Syn, but negative staining for CgA; whereas case two exhibited positive staining for both Syn and CgA.

While the general diagnosis of hepatic NEC normally relies on unique imaging characteristics, IHC markers, and biopsy, the primary hepatic origin of the NEC is normally determined by excluding evidence of origins from other organs [28] given that most hepatic NECs originate from other tissues and later metastasize to the liver [29]. Therefore gastroscopy, colonoscopy, and chest CT including PET-CT are used to finalize the diagnosis of the hepatic origin of NEC [30]. We diagnosed both cases with PHNEC accordingly.

The possibility of co-occurrence of HCC and PHNEC is extremely rare, and we found only 12 cases in the English literature [31–34] and one in the Korean literature [35]. Interestingly, all 12 cases in the English literature are male patients in the age distributions of 40 s (one case), 50 s (2 cases), 60 s (4 cases), and 70 s (5 cases), and the Korean literature reported a case of a 68-year-old female. Almost all patients (11 out of 13) had documented liver disease, chronic hepatitis C (6 cases), chronic hepatitis B (4 cases), and cirrhosis of unknown cause (one case). Our patients were both males around 60 years of age, and case one had hepatitis B and was positive for the HCC marker HepPar-1, suggesting the possibility of co-occurring HCC and PHNEC. Due to the lack of autopsy samples, we cannot absolutely exclude this possibility.

While case two presented with symptoms of yellow skin and sclera, as well as fever and rash during hospitalization, suggesting a diagnosis of autoimmune liver disease, we excluded this possibility after performing liver biopsy. We concluded that the yellow skin and sclera were caused by obstructive jaundice, suggested by an elevated level of total bilirubin with an increased direct bilirubin and normal indirect bilirubin, which is consistent with the imaging results from enhanced-CT that revealed dilation of the bile duct. However, the degree of dilation of the extrahepatic bile duct seemed insufficient to explain the high level of bilirubin. Thus, we concluded that intrahepatic obstructive jaundice arose as a consequence of the neuroendocrine carcinoma having invaded intrahepatic small bile ducts, leading to severe small bile duct obstruction and high levels of direct bilirubin. Case two indicates that yellow skin and sclera from obstructive jaundice are clinical manifestations arising as a consequence of bile duct invasion by neuroendocrine carcinoma. Thus, jaundice can be consistent with diagnosis of neuroendocrine carcinoma having originated in the liver and metastasized to the bile duct. However, we could not rule out the possibility of cholangiocarcinoma or independent primary origin of biliary NEC in addition to PHNEC, and it is nearly impossible to distinguish between biliary NEC and cholangiocarcinoma without biopsy or autopsy [36]. As for case one, since we did not see either lesions of the bile duct through multiple imaging approaches or symptoms suggesting obstruction of the bile duct, we excluded the possibility of cholangiocarcinoma or biliary NEC.

Furthermore, the PHNEC of case two was found to be bilobar, with tumors in both lobes of the liver, suggesting the multifocality of the primary. One review reported that 37.2% (35 out of 94) of PHNEC cases had multiple tumors involved, and 23.4% (22 out of 94) PHNEC were bilobar [7]; another review reported that 23.7% (28 out of 118 cases) of PHNEC were ‘multicentric’ with a right lobe bias (48.4%, 60/124), and 18.5% (23/124) were bilobar PHNEC [37].

There are multiple treatment options for PHNEC. For early stage PHNEC, surgical resection of liver tumor tissue or partial hepatectomy is the most common treatment [7], with a five-year survival of 74–78% [4]. Otherwise, treatment options are limited to liver transplantation, TACE, or administration of somatostatin [11, 38–42]. Neither of our two cases was eligible for surgical resection of the tumor, and liver transplantation was not performed due to a lack of matched donor livers or financial concerns. Accordingly, case one received TACE treatment. As TACE was contraindicated for case two, this patient received somatostatin treatment, but his allergic reaction forced us to resort to a supportive therapy of albumin administration to protect the liver.

While the inhibitory hormone somatostatin is useful for alleviating the symptoms of the excessive hormones released by many NECs and inhibiting tumor growth. Particularly, administration of a high dose of the radiolabeled somatostatin analogue ^111^indium-octreotide, which is typically used to detect neuroendocrine tumors, may hold promise as a therapeutic agent [43, 44]. However, it has been reported that PHNEC is normally endocrinologically silent (i.e. the tumors do not present with carcinoid syndrome) due to the rapid degradation of neoplastic-derived hormones via portal circulation, and only 6.8% (6 out of 88 cases) of PHNEC presented with classic carcinoid syndrome [37], although another review reported slightly higher 16.7% (14 out of 84) carcinoid syndrome-presenting cases [7]. Consistently, neither of our cases presented classic carcinoid syndrome, and case two was allergic to somatostatin analogue octreotide. Thus, somatostatin analogues have not been demonstrated to be therapeutically effective in the treatment of PHNEC [45, 46], and the potential for allergic reaction to somatostatin analogues should be anticipated.

Targeted therapies such as treatment with mTOR inhibitor (everolimus) or tyrosine kinase inhibitor (ranitidine) have shown promise for the treatment of advanced pancreatic neuroendocrine tumors [47–49]. Everolimus is currently approved by the FDA for targeted therapy of well-differentiated, nonfunctional neuroendocrine tumors of gastrointestinal origin [50]. However, for PHNEC, there are no approved targeted therapies available [51].

The prognosis of PHNEC depends on the size of the tumor, the degree of differentiation (well, moderately or poorly differentiated), histologic grade, Ki-67 index, and status of metastasis [37, 52–54]. Currently, the overall prognosis of PHNEC is better than other types of liver cancer [41, 55]. Median survival is 16.5 months (range, 0.7 to 41.7 months) based on a review of 12 PHNEC patients [1]. The five-year survival following surgery for all three differentiation subtypes of PHNEC is about 75% [37]. After surgical resection, PHNEC can recur or metastasize in one to 10 years [1, 56, 57]. For poorly differentiated NEC, the five-year survival rate is only 6% [52]. Both cases we reported exhibited the most aggressive and malignant grade 3 poorly differentiated PHNEC with a high Ki-67 index, surviving only 61 and 109 days, respectively, after initial hospital admission.

## Conclusions

Due to the rarity and asymptomatic clinical features of PHNEC, the diagnosis of PHNEC is very challenging. Imaging approaches such as CT and MRI firstly suggest an initial diagnosis of liver cancer. IHC staining of liver biopsies can then lead to an accurate diagnosis of NEC, and subsequent confirmation of hepatic origin of NEC can be achieved by using gastroscopy, colonoscopy and PET-CT examinations to rule out non-hepatic primary sources of the tumor. Survival benefits from treatment of TACE and somatostatin analogues may be limited, and the potential for allergic reaction to somatostatin analogues should be anticipated. Overall, the prognosis of poorly differentiated grade 3 small-cell PHNEC is poor due to limited and ineffective treatment options.

## Additional files

Additional file 1: **Figure S1.** Microscopic finding of a carcinoma of neuroendocrine cells in the liver biopsy from case one, the original image of Fig. 5, before adjustment in Photoshop to remove the pink background and increase the contrast. (PDF 3304 kb)

Additional file 2: **Figure S2.** Microscopic finding of a carcinoma of neuroendocrine cells in the liver biopsy from case two, the original image of Fig. 8 before adjustment in Photoshop to remove the pink background and increase the contrast. (PDF 3192 kb)

## Abbreviations

AFP: Alpha-fetoprotein
ALT: Alanine aminotransferase
AST: Aspartate aminotransferase
CA: Cancer antigen
CgA: Chromogramin A
CI: Confidence interval
CT: Computer tomography
DCE-MRI: Dynamic contrast-enhanced magnetic resonance imaging
HCC: Hepatocellular carcinoma
IHC: Immunohistochemical
NEC: Neuroendocrine carcinomas
NSE: Neuron specific enolase
PET-CT: Positron emission tomography-computer tomography
PHNEC: Primary hepatic neuroendocrine carcinoma
SF: Serum ferritin
Syn: Synaptophysin
TACE: Transcatheter arterial chemoembolization
TB: Total bilirubin

## References

[1] Park CH, Chung JW, Jang SJ, Chung MJ, Bang S, Park SW, Song SY, Chung JB, Park JY (2012). Clinical features and outcomes of primary hepatic neuroendocrine carcinomas. J Gastroenterol Hepatol.

[2] Shetty PK, Baliga SV, Balaiah K, Gnana PS (2010). Primary hepatic neuroendocrine tumor: an unusual cystic presentation. Indian J Pathol Microbiol.

[3] Skagias L, Vasou O, Ntinis A, Kondi-Pafiti A, Koureas A, Politi E (2010). Primary hepatic neuroendocrine tumor with exophytic growth: report of a case with diagnosis by fine needle aspiration biopsy. Acta Cytol.

[4] Camargo ES, Viveiros Mde M, Correa Neto IJ, Robles L, Rezende MB (2014). Primary hepatic carcinoid tumor: case report and literature review. Einstein (Sao Paulo).

[5] Oronsky B, PC Ma, Morgensztern D, Carter CA. Nothing But NET: A Review of Neuroendocrine Tumors and Carcinomas. Neoplasia. 2017;19(12):991–1002. 10.1016/j.neo.2017.09.002. 10.1016/j.neo.2017.09.002 PMC5678742 29091800

[6] Quartey B (2011). Primary hepatic neuroendocrine tumor: what do we know now?.

[7] Lin CW, Lai CH, Hsu CC, Hsu CT, Hsieh PM, Hung KC, Chen YS (2009). Primary hepatic carcinoid tumor: a case report and review of the literature. Cases J.

[8] Knox CD, Anderson CD, Lamps LW, Adkins RB, Pinson CW (2003). Long-term survival after resection for primary hepatic carcinoid tumor. Ann Surg Oncol.

[9] Zhang A, Xiang J, Zhang M, Zheng S (2008). Primary hepatic carcinoid tumours: clinical features with an emphasis on carcinoid syndrome and recurrence. J Int Med Res.

[10] Akahori T, Sho M, Tanaka T, Nishiofuku H, Kinoshita S, Nagai M, Kichikawa K, Nakajima Y (2013). Significant efficacy of new transcatheter arterial chemoembolization technique for hepatic metastases of pancreatic neuroendocrine tumors. Anticancer Res.

[11] Bencsikova B (2016). Antiproliferative effect of somatostatin analogs - data analyses and clinical applications in the context of the CLARINET study. Klinicka onkologie.

[12] Chamberlain RS, Canes D, Brown KT, Saltz L, Jarnagin W, Fong Y, Blumgart LH (2000). Hepatic neuroendocrine metastases: does intervention alter outcomes?. J Am Coll Surg.

[13] Iwao M, Nakamuta M, Enjoji M, Kubo H, Fukutomi T, Tanabe Y, Nishi H, Taguchi KI, Kotoh K, Nawata H (2001). Primary hepatic carcinoid tumor: case report and review of 53 cases. Med Sci Monit.

[14] Donadon M, Torzilli G, Palmisano A, Del Fabbro D, Panizzo V, Maggioni M, Santambrogio R, Montorsi M (2006). Liver resection for primary hepatic neuroendocrine tumours: report of three cases and review of the literature. Eur J Surg Oncol.

[15] Huang YQ, Xu F, Yang JM, Huang B (2010). Primary hepatic neuroendocrine carcinoma: clinical analysis of 11 cases. Hepatobiliary Pancreat Dis Int.

[16] Rückert R, Rückert J, Dörffel Y, Rudolph B, Müller J (1999). Primary hepatic neuroendocrine tumor: successful hepatectomy in two cases and review of the literature. Digestion.

[17] Akahoshi T, Higashi H, Tsuruta S, Tahara K, Matsumoto T, Takeuchi H, Era S, Fujita F, Muto Y (2010). Primary neuroendocrine carcinoma coexisting with hemangioma in the liver: report of a case. Surg Today.

[18] Modlin IM, Lye KD, Kidd M (2003). A 5-decade analysis of 13,715 carcinoid tumors. Cancer.

[19] Nikfarjam M, Muralidharan V, Christophi C (2004). Primary hepatic carcinoid tumours. HPB (Oxford).

[20] Morishita A, Yoneyama H, Nomura T, Sakamoto T, Fujita K, Tani J, Miyoshi H, Haba R, Masaki T (2016). Primary hepatic neuroendocrine tumor: a case report. Mol Clin Oncol.

[21] Kellock T, Tuong B, Harris AC, Yoshida E (2014). Diagnostic imaging of primary hepatic neuroendocrine tumors: a case and discussion of the literature. Case Rep Radiol.

[22] Elsayes KM, Menias CO, Bowerson M, Osman OM, Alkharouby AM, Hillen TJ (2011). Imaging of carcinoid tumors: spectrum of findings with pathologic and clinical correlation. J Comput Assist Tomogr.

[23] Hasegawa H, Kuzushita N, Nakazuru S, Itoh M, Araki M, Yoshioka C, Suemura S, Ohta M, Yoshio T, Toyama T (2010). Case of primary hepatic neuroendocrine carcinoma diagnosed by needle biopsy. Nihon Shokakibyo Gakkai Zasshi.

[24] Tamm EP, Kim EE, Ng CS (2007). Imaging of neuroendocrine tumors. Hematol Oncol Clin North Am.

[25] Wang L-X, Liu K, Lin G-W, Jiang T (2015). Primary hepatic neuroendocrine tumors: comparing CT and MRI features with pathology. Cancer Imaging.

[26] Pilichowska M, Kimura N, Ouchi A, Lin H, Mizuno Y, Nagura H (1999). Primary hepatic carcinoid and neuroendocrine carcinoma: clinicopathological and immunohistochemical study of five cases. Pathol Int.

[27] Sundin A, Eriksson B, Bergström M, Långström B, Öberg K, Örlefors H (2004). PET in the diagnosis of neuroendocrine tumors. Ann N Y Acad Sci.

[28] Wang LM, An SL, Wu JX. Diagnosis and therapy of primary hepatic neuroendocrine carcinoma: clinical analysis of 10 cases. Asian Pac J Cancer Prev. 2014;15(6):2541–6. https://www.ncbi.nlm.nih.gov/pubmed/24761861. 10.7314/apjcp.2014.15.6.2541 24761861

[29] Ihse I, Persson B, Tibblin S (1995). Neuroendocrine metastases of the liver. World J Surg.

[30] Seemann MD, Meisetschlaeger G, Gaa J, Rummeny EJ (2006). Assessment of the extent of metastases of gastrointestinal carcinoid tumors using whole-body PET, CT, MRI, PET/CT and PET/MRI. Eur J Med Res.

[31] Choi GH, Ann SY, Lee SI, Kim SB, Song IH (2016). Collision tumor of hepatocellular carcinoma and neuroendocrine carcinoma involving the liver: case report and review of the literature. World J Gastroenterol.

[32] Baker E, Jacobs C, Martinie J, Iannitti DA, Vrochides D, Swan RZ (2016). Mixed hepatocellular carcinoma, neuroendocrine carcinoma of the liver. Am Surg.

[33] Okumura Y, Kohashi K, Wang H, Kato M, Maehara Y, Ogawa Y, Oda Y (2017). Combined primary hepatic neuroendocrine carcinoma and hepatocellular carcinoma with aggressive biological behavior (adverse clinical course): a case report. Pathol Res Pract.

[34] Lu JG, Farukhi MA, Mayeda D, French SW (2017). Hepatocellular carcinoma with neuroendocrine differentiation: a case report. Exp Mol Pathol.

[35] Yun EY, Kim TH, Lee SS, Kim HJ, Kim HJ, Jung WT, Lee OJ, Song DH (2016). A case of composite hepatocellular carcinoma and neuroendocrine carcinoma in a patient with liver cirrhosis caused by chronic hepatitis B. Korean J Gastroenterol.

[36] Michalopoulos N, Papavramidis TS, Karayannopoulou G, Pliakos I, Papavramidis ST, Kanellos I (2014). Neuroendocrine tumors of extrahepatic biliary tract. Pathol Oncol Res.

[37] Quartey B (2011). Primary hepatic neuroendocrine tumor: what do we know now?. World J Surg Oncol.

[38] Mehta DC, Warner RRP, Parnes I, Weiss M (1996). An 18-year follow-up of primary hepatic carcinoid with carcinoid syndrome. J Clin Gastroenterol.

[39] Sano K, Kosuge T, Yamamoto J, Shimada K, Takayama T, Yamasaki S, Makuuchi M (1998). Primary hepatic carcinoid tumors confirmed with long-term follow-up after resection. Hepato-Gastroenterology.

[40] Iimuro Y, Deguchi Y, Ueda Y, Tanaka A, Iwasa Y, Ishihara M, Mizuta K, Yamamoto Y, Ikai I, Shimahara Y (2002). Primary hepatic carcinoid tumor with metachronous lymph node metastasis after long-term follow up. J Gastroenterol Hepatol.

[41] Fenwick SW, Wyatt JI, Toogood GJ, Lodge JP (2004). Hepatic resection and transplantation for primary carcinoid tumors of the liver. Ann Surg.

[42] de Liguori CN, Manzia T, Tariciotti L, Berlanda M, Orlando G, Tisone G. Liver transplantation in primary hepatic carcinoid tumor: case report and literature review. In: Transplantation proceedings: 2009: Elsevier; 2009. p. 1386–9. http://www.transplantation-proceedings.org/article/S0041-1345(09)00398-4/fulltext. 10.1016/j.transproceed.2009.03.011 19460567

[43] Weiner RE, Thakur ML (2002). Radiolabeled peptides in the diagnosis and therapy of oncological diseases. Appl Radiat Isot.

[44] Bodei L, Kwekkeboom DJ, Kidd M, Modlin IM, Krenning EP (2016). Radiolabeled somatostatin analogue therapy of Gastroenteropancreatic cancer. Semin Nucl Med.

[45] Yang K, Cheng YS, Yang JJ, Jiang X, Guo JX (2015). Primary hepatic neuroendocrine tumor with multiple liver metastases: a case report with review of the literature. World J Gastroenterol.

[46] Sarmiento JM, Heywood G, Rubin J, Ilstrup DM, Nagorney DM, Que FG (2003). Surgical treatment of neuroendocrine metastases to the liver: a plea for resection to increase survival. J Am Coll Surg.

[47] Jensen RT, Delle Fave G (2011). Promising advances in the treatment of malignant pancreatic endocrine tumors. N Engl J Med.

[48] Yao JC, Shah MH, Ito T, Bohas CL, Wolin EM, Van Cutsem E, Hobday TJ, Okusaka T, Capdevila J, de Vries EG (2011). Everolimus for advanced pancreatic neuroendocrine tumors. N Engl J Med.

[49] Raymond E, Dahan L, Raoul JL, Bang YJ, Borbath I, Lombard-Bohas C, Valle J, Metrakos P, Smith D, Vinik A (2011). Sunitinib malate for the treatment of pancreatic neuroendocrine tumors. N Engl J Med.

[50] Chan DL, Segelov E, Singh S (2017). Everolimus in the management of metastatic neuroendocrine tumours. Ther Adv Gastroenterol.

[51] Mulvey CK, Bergsland EK (2016). Systemic therapies for advanced gastrointestinal carcinoid tumors. Hematol Oncol Clin North Am.

[52] Hentic O, Couvelard A, Rebours V, Zappa M, Dokmak S, Hammel P, Maire F, O'Toole D, Lévy P, Sauvanet A (2011). Ki-67 index, tumor differentiation, and extent of liver involvement are independent prognostic factors in patients with liver metastases of digestive endocrine carcinomas. Endocr Relat Cancer.

[53] Bosman FT, Carneiro F, Hruban RH, Theise ND (2010). WHO classification of Tumours of the digestive system.

[54] Yao JC, Hassan M, Phan A, Dagohoy C, Leary C, Mares JE, Abdalla EK, Fleming JB, Vauthey JN, Rashid A (2008). One hundred years after “carcinoid”: epidemiology of and prognostic factors for neuroendocrine tumors in 35,825 cases in the United States. J Clin Oncol.

[55] Yau T, Yao TJ, Chan P, Epstein RJ, Ng KK, Chok SH, Cheung TT, Fan ST, Poon RT (2009). The outcomes of elderly patients with hepatocellular carcinoma treated with transarterial chemoembolization. Cancer.

[56] Schwartz G, Colanta A, Gaetz H, Olichney J, Attiyeh F (2008). Primary carcinoid tumors of the liver. World J Surg Oncol.

[57] Srivastava A, Hornick JL (2009). Immunohistochemical staining for CDX-2, PDX-1, NESP-55, and TTF-1 can help distinguish gastrointestinal carcinoid tumors from pancreatic endocrine and pulmonary carcinoid tumors. Am J Surg Pathol.

