# Supplementary figures and images for: Primary hepatic neuroendocrine carcinoma: report of two cases and literature review
Source: BMC Clin Pathol. 2018 Mar 1;18:3. doi: 10.1186/s12907-018-0070-7 (PMC5831736; doi:10.1186/s12907-018-0070-7)

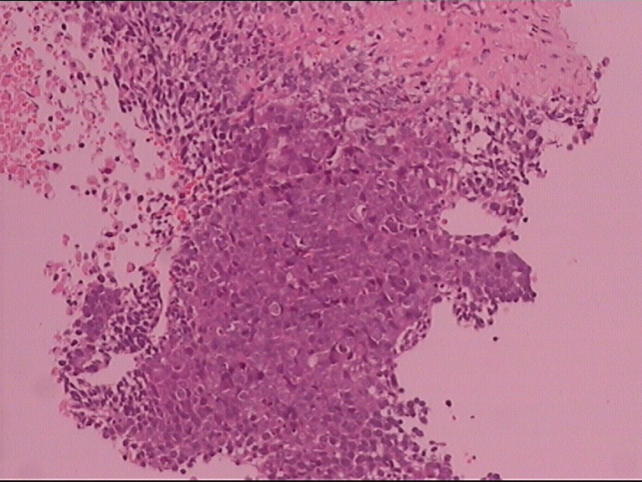

Supplement: Supplementary file 1 — Figure S1. Microscopic finding of a carcinoma of neuroendocrine cells in the liver biopsy from case one, the original image of Fig. 5, before adjustment in Photoshop to remove the pink background and increase the contrast. (PDF 3304 kb) [file 12907_2018_70_MOESM1_ESM.pdf]

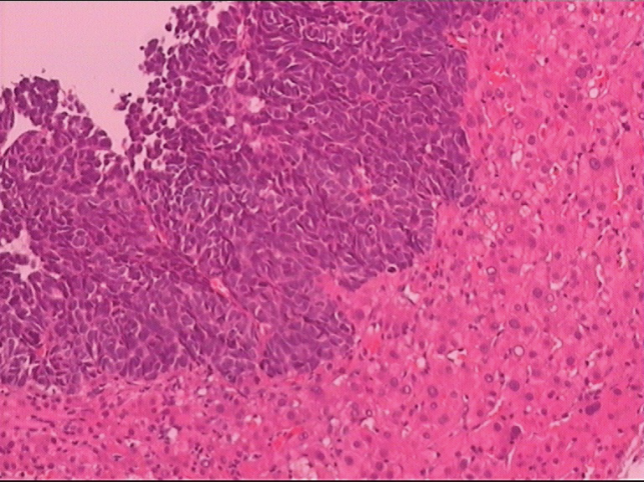

Supplement: Supplementary file 2 — Figure S2. Microscopic finding of a carcinoma of neuroendocrine cells in the liver biopsy from case two, the original image of Fig. 8 before adjustment in Photoshop to remove the pink background and increase the contrast. (PDF 3192 kb) [file 12907_2018_70_MOESM2_ESM.pdf]
